# Hepatopulmonary Syndrome with Right-to-left Shunt in Cirrhotic Patients Using Macro-Aggregated Albumin Lung Perfusion Scan: Comparison with Contrast Echocardiography and Association with Clinical Data

**DOI:** 10.4274/mirt.galenos.2019.30301

**Published:** 2020-02-17

**Authors:** Zeynab Alipour, Abbas Armin, Sudabeh Mohamadi, Seyed Masoud Tabib, Zahra Azizmohammadi, Ali Gholamrezanezhad, Majid Assadi

**Affiliations:** 1 Bushehr University of Medical Sciences, Bushehr Medical Center Hospital, Department of Internal Medicine, Division of Gastroenterology, Bushehr, Iran; 2 Bushehr University of Medical Sciences, Faculty of Medicine, Department of Community Medicine, Bushehr, Iran; 3 Shahid Beheshti University of Medical Sciences, Imam Hossein Hospital, Department of Nuclear Medicine, Tehran, Iran; 4 University of Southern California, Keck School of Medicine, Department of Diagnostic Radiology, Los Angeles, USA; 5 Bushehr University of Medical Sciences, Bushehr Medical University Hospital, The Persian Gulf Nuclear Medicine Research Center, Department of Molecular Imaging and Radionuclide Therapy (MIRT), Bushehr, Iran

**Keywords:** Hepatopulmonary syndrome, cirrhosis, macro-aggregated albumin lung perfusion scan, contrast echocardiography

## Abstract

**Objectives:**

The diagnosis of hepatopulmonary syndrome (HPS) which is a common complication in cirrhotic patients is still subject to debate. This study investigated the association of clinical findings with HPS in cirrhotic patients using macro-aggregated albumin lung perfusion scan (^99m^Tc-MAA lung scintigraphy). In addition, comparison between ^99m^Tc-MAA lung scintigraphy and contrast echocardiography (CEE) in detection of HPS was also performed.

**Methods:**

In this study, 27 patients with cirrhosis underwent ^99m^Tc-MAA lung scintigraphy and contrast echocardiography comparison CEE and the frequency of HPS was assessed in them and also was compared across the other variables.

**Results:**

The ^99m^Tc-MAA lung scintigraphy showed HPS in 13 patients (48.1%) while CEE demonstrated HPS in 5 patients with cirrhosis (18.51%). HPS was mild in 40.74% (11/27) of the patients, and severe in only 2 patients. There was no relationship between gender, disease duration, having diagnosis of disease previously, pulmonary symptoms and Child-Pugh score variations and HPS (p>0.05). Comparison of hemodynamic indices, arterial blood gas analysis and laboratory indices between patients with and without HPS was also non-significant (p value >0.05). Among coagulation factors assessed in cirrhotic patients, we found only significant correlation between HPS and prothrombin time (p<0.05).

**Conclusion:**

HPS, particularly its mild form, is noted in a great number of patients with cirrhosis using 99mTc-MAA lung scintigraphy. Because of its technical ease, and possibility to obtain objective quantitative information, ^99m^Tc-MAA lung scintigraphy can be complementary to other diagnostic methods in the evaluation of HPS assessment, although additional studies are needed.

## Abstract (tr)

**Amaç:**

Sirotik hastalarda sık görülen bir komplikasyon olan hepatopulmoner sendromun (HPS) tanısı halen tartışmaya açıktır. Bu çalışmada, makroagregat albümin akciğer perfüzyon sintigrafisi (^99m^Tc-MAA akciğer sintigrafisi) kullanılarak sirotik hastalarda klinik bulgularla HPS’nin ilişkisi araştırılmıştır. Ayrıca, HPS’nin saptanmasında ^99m^Tc-MAA akciğer sintigrafisi ile kontrast ekokardiyografi (KE) karşılaştırılmıştır.

**Yöntem:**

Bu çalışmada, sirozlu 27 hastada ^99m^Tc-MAA akciğer sintigrafisi ve KE karşılaştırıldı ve HPS sıklığı değerlendirildi. Ayrıca HPS’nin sıklığı ile diğer değişkenler arasındaki ilişki araştırıldı.

**Bulgular:**

^99m^Tc-MAA akciğer sintigrafisi 13 sirozlu hastada (%48,1) HPS’nin varlığını gösterirken, KE 5 sirozlu hastada (%18,51) gösterdi. HPS hastaların %40,74’inde (11/27) hafif, sadece 2 hastada şiddetliydi. Cinsiyet, hastalık süresi, hastalık geçmişi, akciğer semptomları ve Child skoru ile HPS arasında herhangi bir ilişki yoktu (p>0,05). Hemodinamik indeksler, arteriyel kan gazı analizi ve laboratuvar indeksleri açısından HPS’li hastalar ve HPS’li olmayan hastalar arasında fark görülmedi (p>0,05). Sirotik hastalarda değerlendirilen koagülasyon faktörleri arasında, sadece HPS ve protrombin zamanı arasında anlamlı bir korelasyon bulundu (p<0,05).

**Sonuç:**

^99m^Tc-MAA akciğer sintigrafisi ile çok sayıda sirozlu hastada HPS’nin özellikle hafif formu saptandı. Teknik kolaylığı ve objektif kantitatif bilgi edinme olasılığı nedeniyle, ^99m^Tc-MAA akciğer sintigrafisi HPS’nin değerlendirilmesinde diğer tanı yöntemlerini tamamlayıcı olabilir, ancak ek çalışmalara ihtiyaç vardır.

## Introduction

Cirrhosis is a pathologic process in which normal liver structure is substituted by scar tissue. Cirrhotic patients are vulnerable to many side effects that reduce their lifetime. One of these side effects is hypoxia resulting from hepatopulmonary syndrome (HPS) (1,2,3).

HPS, which can influence patient’s prognosis, is described by a clinical triad entailing being of late stage liver disease, gas exchange disorders, eventually leading to hypoxemia and the occurrence of intrapulmonary vascular dilatations (IPVD), without being of intrinsic pulmonary disease (4).

Many conditions can influence the gas exchange in lungs (5). Ascites, pleural effusion, hepatomegaly and basal lung lobes atelectasis are the most common identified causes and can disturb oxygen exchange in a restrictive manner. On the other hand, some side effects of cirrhosis such as HPS or portopulmonary hypertension may not be diagnosed by physical examination, pulmonary imaging modalities or pulmonary function tests (6).

In addition, increased mortality rate of cirrhotic patients due to HPS is reported (6).

The only method to cure this status is liver transplantation, so, early diagnosis of this status carries important clinically significance (6).

The term HPS was coined by Kennedy and Knudson in 1977 (7,8). Signs of HPS are dyspnea, platypnea and orthodeoxia and HPS is diagnosed with a clinical triad including chronic liver disease, increased alveolar-arterial gradient of O2 [p (A-a) O2] ≥15 mmHg (≥20 mmHg for patients over 64 years) and the presence of intrapulmonary right to left shunt (9,10,11).

The prevalence of this syndrome has not been completely understood because figures depend on the manner used for the identification and the characteristics of the population investigated (12,13).

The intrapulmonary arteriovenous shunt can be diagnosed by contrast enhanced echocardiography (CEE) and ^99m^Tc-labelled macro aggregated albumin scintigraphy (^99m^Tc-MAA).

Contrast CEE is considered the standard technique (14) which in this method, a liquid with bubbles is injected into a peripheral vein and the liquid entering the left cavities is observed withsaline bubble test.

In ^99m^Tc-labelled macro aggregated albumin scintigraphy (^99m^Tc-MAA), ^99m^Tc labelled albumin particles are injected into a peripheral vein and then are capable to reach extra pulmonary sites like brain or kidneys parenchyma due to the presence of IPVD and intra pulmonary arteriovenous shunt (14,15,16) however, there is a doubt that in view of predisposing vasoconstriction in brain and kidney of cirrhotic patients, ^99m^Tc-MAA scan may not be reliable in this setting (17).

In current study, we evaluated cirrhotic patients to find the frequency of HPS in these patients with assistance of clinical and paraclinical methods.

## Materials and Methods

In this study, we evaluated 27 cirrhotic patients referred to the Department of Nuclear Medicine of a University Affiliated Hospital between 2017-2018.

Patients were divided into three groups based on Child-pugh and Meld scoring systems.  The Child-pugh score employs five clinical measures (including total serum bilirubin, serum albumin, prothrombin time (PTT), ascites and hepatic encephalopathy) of liver disease. Each measure is scored 1-3, with 3 indicating most severe derangement. These three groups were as follow: group A (5-7), group B (8-9) and group C (10-15).

The Meld score is also calculated using a mathematical formula that is based on three laboratory results including total serum bilirubin, INR and SCr.

MELD formula= 3.78×ln [serum bilirubin (mg/dL)] + 11.2×ln (INR) + 9.57×ln [serum creatinine (mg/dL)] + 6.43.

Furthermore, we collected each patient`s clinical history of dyspnea and other pulmonary symptoms and we accepted the presence of intrapulmonary arterio venous shunt >6% as abnormal finding and used it for HPS diagnosis.

During the admission time, lung perfusion scintigraphy,  echocardiography or spirometry, and laboratory tests including arterial blood gas (ABG), complete blood count and liver function tests were carried out and above mentioned results were compared with patient’s age, arterio venous shunt presence and severity of pulmonary failure. As mentioned above, HPS abundance was calculated in these cirrhotic patients.

The radionuclide study was carried out by injecting 1-4 mCi ^99m^Tc-MAA intravenously. In cases of intrapulmonary shunting as in HPS, some amount of radiotracers goes through the lungs into the systemic circulation like brain, kidneys and thyroid along with the lung. The pulmonary shunt percent is calculated by applying the geometric mean (GM) of brain and lung counts in the formula:

(GM brain) / (GM brain + GM lung ) *100 (normal <6%)

Contrast enhanced bubble CEE  was done by agitating a small amount of air with saline to produce bubbles using a three-way stopcock, when administered into the venous circulation. Appearing of even one bubble in the left side of the heart has been considered as a criterion of right-to left shunting.

Moreover, all eligible participants signed an inform consent. This study complies with the Declaration of Helsinki, and it was confirmed by the Ethics Committee of Bushehr University of Medical Sciences.

### Statistical Analysis

Categorical variables were analyzed using chi-square test and continuous variables using Student’s t-test. Categorical values were expressed as percentage and continuous values were expressed as mean value ± standard deviation. Linear regression analysis was used to determine whether there was a correlation between above mentioned findings and HPS. P value <0.05 was considered as statically significant for all statistical tests. Statistical analysis was performed with the use of the SPSS statistical package (version 24).

## Results

Our studied cirrhotic population consisted of 18 males (66.6%) and 9 females (33.3%), with mean age of 52.3±17.28 years. From this population, 13 patients (10 males and 3 females) had HPS due to the results of ^99m^Tc-MAA scintigraphy, therefore, 48.15% had HPS and 51.85% did not have HPS. HPS was mild in 40.74% (11/27) of the patients, and severe in only 2 patients.

In comparison, CEE demonstrated five positive shunts (18.51%) and the remaining did not have shunt.

The average age of the patients with HPS was 45.5±13.43 years and it was 58.7±18.46 years in the patients without HPS. There was no statistically significant difference between groups in terms of age (p>0.05).

There was no relationship between gender, disease duration, having diagnosis of disease previously, pulmonary symptoms (dyspnea, ortodeoxia, plathypnea and orthopnea) Child-pugh score variations and HPS (Table 1) (p>0.05).

Comparison of hemodynamic indices, ABG analysis and laboratory indices between patients with and without HPS was also non-significant (p>0.05) (Table 2,3,4).

Among coagulation factors assessed in cirrhotic patients, we found only significant (p<0.05) correlation between HPS and PTT (Table 5).

**Figure 1:**
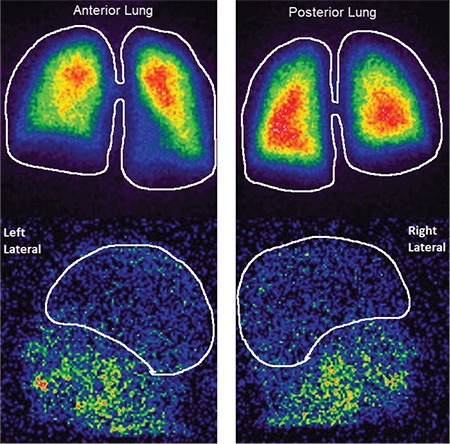
Normal scanning: ^99m^Tc-MAA is only accumulated in the lungs (shunt=1%)

**Figure 2:**
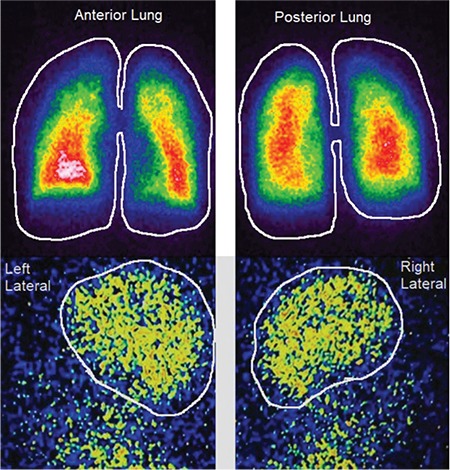
Hepatopulmonary syndrome: ^99m^Tc-MAA is accumulated in the lungs, and brain (shunt=26%)

Two images of positive and negative scans for HPS are illustrated in Figure 1 and 2.

## Discussion

HPS is one of the most important problems in cirrhotic patients. In this study, 13 (48.1%) cases with established HPS were detected. Moreover, there was significant correlation between PTT and HPS  in current study (p<0.05).

Surasi et al. (14) concluded that the ^99m^Tc-MAA lung perfusion scintigraphy was helpful and could diagnose HPS in cirrhotic patients by finding the intrapulmonary arteriovenous shunt.

El-Shabrawi et al. (15) compared the findings of contrast CEE and ^99m^Tc-MAA lung perfusion scintigraphy in 40 children with chronic liver disease and showed that lung perfusion scintigraphy with ^99m^Tc-MAA was more sensitive than contrast CEE for determining intrapulmonary arteriovenous shunt in the patients with chronic hepatic failure. The result of that study was the same as with current study, in which a statistically significant difference between these two methods was noted.

In the current report seven patients (48.15%) demonstrated right to left intrapulmonary shunts shown by lung perfusion scintigraphy. HPS was mild in 40.74% (11/27) of the patients, and severe in only 2 patients.  This may be most likely due to the point that perfusion scintigraphy is able to identify trivial shunts using quantitative analysis, so CEE might fail to detect small shunts. Furthermore, CEE is also operator reliant.

In contrary, there are a few reports showing that CEE is more sensitive than lung perfusion scintigraphy for the diagnosis of intrapulmonary shunting (9). In addition, CEE can be done as a part of standard echocardiographic screening for pulmonary hypertension and the European Respiratory Society Task Force on Pulmonary-hepatic vascular diseases has advised CEE as the first step method in screening of HPS (8).

In another study done by Grimon et al. (17), 135 children with chronic hepatic failure were evaluated and they delineated that ^99m^Tc-MAA scintigraphy was more accurate than ABG analysis in detection of intrapulmonary arteriovenous shunt. Although we found the same result, there was no correlation between ABG indices and HPS.

Likewise, Fatemi et al. (18) worked on 54 cirrhotic patients and showed that 10 patients had the clinical criteria of HPS and 7 patients had sub-clinical criteria of HPS. Their rate was  less than our rate of 48%. In their study, the most prevalent clinical signs were dyspnea and cyanosis. Dyspnea had high sensitivity and achropachy had high specificity in cirrhotic patients with HPS.

On the other hand , PO_2_<70 and alveolar-arterial gradient had the highest sensitivity in this era (19) however, we did not find any relationship between these laboratory indices and HPS.

Fragaki et al. (20) assessed HPS in cirrhotic patients using ^99m^Tc-MAA lung scintigraphy and correlated the results with clinical data. In total, 94 out of 102 included patients had complete scintigraphic data. Overall, 24 (26%) patients had HPS and 95.8% of them had mild-to-moderate HPS. There was no significant difference in terms of HPS between decompensated (24.6%) and compensated cirrhosis (27.3%). In the multivariate analysis, only the quantitative index was noteworthy for the identification of HPS. They concluded that mild-to moderate HPS had no substantial effect on survival of cirrhotic patients. Also in our study, most of the detected HPS was mild (40.74%).

In contrast to our finding, Grilo et al. (21) who assessed ^99m^Tc-MAA lung perfusion scan in 115 cirrhotic subjects with HPS candidates for liver transplantation, demonstrated that the ^99m^Tc-MAA had a low sensitivity  for the diagnosis of HPS.

However, it should be noted that ^99m^Tc-MAA lung scintigraphy as compared with CEE, has  disadvantages of underestimation of intrapulmonary shunt fraction in advanced liver disease because of renal and cerebral arterial vasoconstriction occurring  in patients with cirrhosis which increases with progression of the liver disease (16). In addition, ^99m^Tc-MAA lung scintigraphy depicts total value of right to left shunt, which may be due to cardiac problem in origin, so performance of CEE can assess one-stop shop.

This study suffered from some demerits; the most important ones were a small overall sample of participating patients, and lack of follow up to assess the survival analysis. Therefore, further research necessitates a larger number of patients split into more categories of HPS to find the best clinical outcomes.

## Conclusion

HPS, particularly its mild form is noted in a great number of patients with cirrhosis using ^99m^Tc-MAA lung scintigraphy. Because of its technical ease, and possibility to obtain objective quantitative information, ^99m^Tc-MAA lung scintigraphy can be complementary to other diagnostic methods in the evaluation of HPS assessment, although additional studies are needed.

## Figures and Tables

**Table 1:**
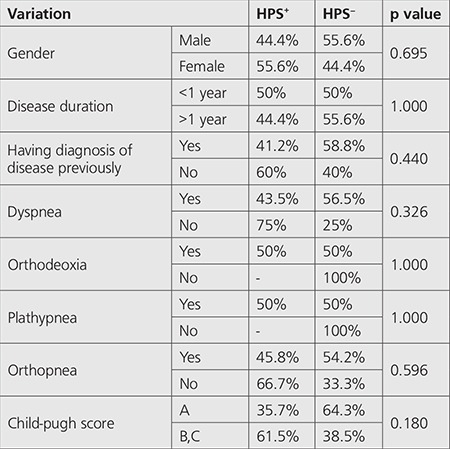
Comparison between gender, disease duration, having diagnosis of disease previously, pulmonary symptoms (dyspnea, ortodeoxia, plathypnea and orthopnea) and child score variations in two groups of patients with and without hepatopulmonary syndrome

**Table 2:**
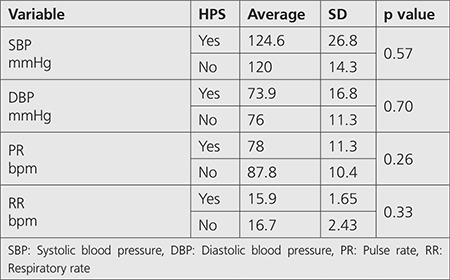
Comparison of hemodynamic indices between patients with and without hepatopulmonary syndrome

**Table 3:**
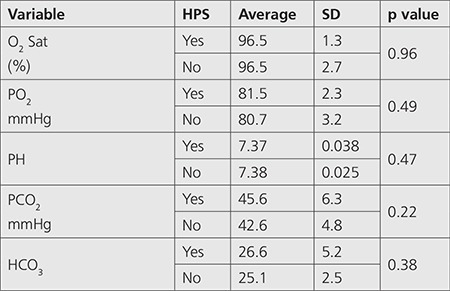
Comparison of arterial blood gas analysis between patients with and without hepatopulmonary syndrome

**Table 4:**
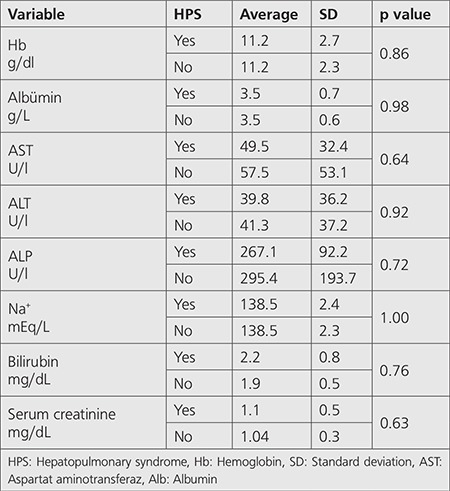
Comparison of laboratory indices between patients with and without hepatopulmonary syndrome

**Table 5:**
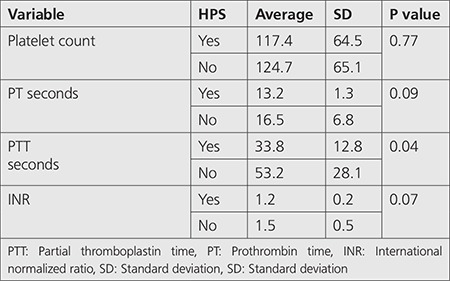
Comparison of coagulation factors between patients with and without hepatopulmonary syndrome
